# Systemic Anti-Inflammatory Agents in the Prevention of Chemoradiation-Induced Mucositis: A Review of Randomised Controlled Trials

**DOI:** 10.3390/biom14050560

**Published:** 2024-05-06

**Authors:** Ali I. Mohammed, Lexi Fedoruk, Nicholas Fisher, Andy Xiaoqian Liu, Samar Khanna, Kaelan Naylor, Ziyi Gong, Antonio Celentano, Mohammad S. Alrashdan, Nicola Cirillo

**Affiliations:** 1 Melbourne Dental School, Faculty of Medicine, Dentistry & Health Sciences, The University of Melbourne, Carlton, VIC 3053, Australia; mohammed.a@unimelb.edu.au (A.I.M.); lfedoruk@student.unimelb.edu.au (L.F.); nffis@student.unimelb.edu.au (N.F.); liuxa@student.unimelb.edu.au (A.X.L.); samark@student.unimelb.edu.au (S.K.); knaylor@student.unimelb.edu.au (K.N.); zggon@student.unimelb.edu.au (Z.G.); antonio.celentano@unimelb.edu.au (A.C.); 2 Department of Oral and Craniofacial Health Sciences, College of Dental Medicine, University of Sharjah, Sharjah 27272, United Arab Emirates; malrashdan@sharjah.ac.ae; 3 Department of Oral Medicine and Oral Surgery, Faculty of Dentistry, Jordan University of Science and Technology, Irbid 22110, Jordan; 4 School of Dentistry, University of Jordan, Amman 11942, Jordan

**Keywords:** anti-Inflammatory agents, mucositis, neoplasms, chemoradiotherapy, randomized controlled trials

## Abstract

Mucositis is a pathological condition characterised by inflammation and ulceration of the mucous membranes lining the alimentary canal, particularly in the mouth (oral mucositis) and the gastrointestinal tract. It is a common side effect of cancer treatments, including chemotherapy and radiotherapy, and it is sometimes responsible for treatment interruptions. Preventing mucositis throughout the alimentary tract is therefore crucial. However, current interventions mainly target either oral or gastrointestinal side effects. This review aimed to investigate the use of systemically administered anti-inflammatory agents to prevent mucositis in cancer patients undergoing cancer treatment. PubMed, Ovid, Scopus, Web of Science, WHO ICTRP and ClinicalTrials.gov were screened to identify eligible randomised controlled trials (RCTs). The published literature on anti-inflammatory agents provides mixed evidence regarding the degree of efficacy in preventing/reducing the severity of mucositis in most anticancer treatments; however, sample size continued to be a significant limitation, alongside others discussed. Our review yielded a list of several anti-inflammatory agents that exhibit potential mucositis-preventive effects in cancer patients undergoing cancer treatment, which can be used to inform clinical practice.

## 1. Introduction

While the success of cancer treatment has improved over time and various treatment modalities have been developed, the most often used therapies are surgery, chemotherapy, and radiotherapy [1]. As new medicines and treatment protocols emerge, various side effects must be taken into consideration by clinicians and patients.

### 1.1. Side Effects of Chemotherapy and Radiotherapy

Chemotherapies frequently target and damage DNA, commonly causing secondary malignancies and damage to healthy tissues systemically. As many chemotherapeutics are cleared though the kidneys, renal tissues are exposed to high concentrations of drugs, leading to acute or late nephrotoxicity [2]. The liver is similarly affected, resulting in hepatitis, cholestasis, and steatosis [3]. Neurotoxicity is frequent, with the central nervous system being somewhat protected in comparison to the peripheral nervous system due to the blood–brain barrier, with the resulting effects being numbness, abnormal sensation, and paraesthesia of the extremities [4]. Gastrointestinal toxicities occur in up to 80% of patients receiving chemotherapies, resulting in pain sensation, nausea, infection, diarrhea, and malabsorption of nutrients [5].

Side effects of radiotherapy can be both acute and long-term. Acute radiation damage predominantly involves rapidly proliferating cells such as epithelial cells on the surfaces of the skin or the digestive tract. Radiation damage is apparent when there is inadequate replacement of normal cell turnover by the damaged stem cells [6]. When acute damage fails to heal completely over prolonged periods, especially in tissues with slow turnover such as the brain, kidney, and liver, later consequences including fibrosis, atrophy, vascular damage, and necrosis can occur [7,8].

### 1.2. Mucositis

Mucositis is a painful and debilitating condition characterised by mucosal barrier injury in the form of inflammation, erythema and ulceration of the mucous membranes lining the digestive tract [9]. It can affect the entirety of the gastrointestinal (GI) canal, including the oral cavity (oral mucositis, OM) and the intestinal tract (intestinal mucositis (IM) [10,11]. Mucositis represents a common adverse effect associated with chemotherapy (CT), radiotherapy (RT) and chemoradiotherapy (CRT) treatments. It manifests in approximately 30–40% of patients undergoing CT, 60% of those undergoing RT and a striking 90% of individuals with head and neck cancer (HNC) receiving CRT [12,13]. Notably, within this last category, around 19% will require hospitalization due to the severity of mucositis, subsequently leading to a compromised prognosis as a result of delayed antineoplastic treatment [12]. While CT and RT have similar cellular events leading to mucositis, the biological pathway is different. CT is administered systemically while RT is targeted to a specific area. Chemotherapy-induced mucositis usually develops around day 5–10 after treatment commencing and peaks shortly afterwards, at around 1–2 weeks [12]. Radiotherapy-induced mucositis is more predictable, with its onset often beginning after 2 weeks of treatment and a cumulative radiation dose of 15 Gy, with the highest severity being observed when the dose surpasses 50 Gy [14].

The impact of mucositis on a patient’s quality of life is significant, inducing pain during eating and speech, swallowing difficulties, and impaired nutrient uptake [15,16]. This can lead to malnutrition, dehydration, and weight loss. As such, it limits the body’s capacity to withstand the rigors of cancer treatment [17]. In most cases, the discomfort is so intense that opioid analgesics become necessary. Mucositis can also negatively affect a patient’s adherence to treatment protocols due to symptom-induced emotional and physical distress, resulting in missed appointments and noncompliance [18]. Conspicuous symptoms of mucositis encompass open sores and perforated mucosal lining, which elevate susceptibility to infection, thereby imperilling patient health and introducing delays in cancer treatment [19,20]. Moreover, a multitude of patient-related factors can further exacerbate both the prevalence and severity of cancer treatment-induced mucositis. These include advanced age (>65 years), poor oral hygiene, hypofunction of the salivary glands, poor periodontal health/history, poor nutrition, and comorbidities like diabetes.

#### 1.2.1. Role of Inflammation in the Pathogenesis of Mucositis

The pathogenesis of mucositis is intricated. The initiation phase of mucositis starts due to chemoradiation-induced DNA strand breakage and cell damage. This is followed by the message generation phase, where the transcription factor NF-kB is activated, triggering the increased production of pro-inflammatory cytokines such as IL-1ß and IL-6. Next, TNF- α activates the ceramide and caspase pathways, further increasing pro-inflammatory cytokine production and making up the signalling and amplification phase [21]. The subsequent phase involves ulceration, characterized by a breach in the mucosa, exposing the area to potential secondary bacterial and fungal infections. The concluding stage is the healing phase, initiated after the cessation or completion of cancer treatment. During this phase, cells regenerate, and the host’s normal flora re-establishes itself [22]. The development of chemotherapy-induced mucositis is closely linked to the increased expression of pro-inflammatory cytokines, particularly TNF-α, IL-1β, and IL-6 [23,24,25,26]. Elevated levels of these cytokines serve as valuable indicators of chemotherapy-induced toxicities. Although the outcomes have not been consistently favourable, targeting pro-inflammatory cytokines with anti-inflammatory agents holds promise for mitigating mucositis progression [27]. Therefore, developing preventive strategies to block early inflammatory cascades is a logical approach given the presence of an inflammatory mechanism, thereby warranting the investigation of anti-inflammatory agents for potential prevention or treatment of mucositis.

#### 1.2.2. Mechanism-Based Interventions for Mucositis

A few treatments have shown success in preventing and/or managing OM [28], such as benzydamine mouthwash, a non-steroidal analgesic and anti-inflammatory, which has been shown to reduce the incidence of grade 3 and higher OM in HNC patients receiving RT, with the placebo showing a 2.6-times higher incidence of OM [22]. Palifermin, a recombinant human keratinocyte growth factor, has demonstrated success in reducing the severity, duration and delaying the development of OM in CT- and CRT-treated cancer patients; however, its practical use is limited due to its prohibitive cost [29,30,31]. Furthermore, emerging evidence supports the effectiveness of photobiomodulation (PBM) therapy, also known as low-level laser therapy (LLLT), which has shown promising results in preventing OM in head and neck cancer patients undergoing concurrent chemoradiation therapy [32,33]. Other therapies, including the local application of cryotherapy and PTA (polymyxin E, tobramycin, and amphotericin B), as well as granulocyte macrophage-colony-stimulating factor/granulocyte colony-stimulating factor (GM-CSF/GCSF), show varying degrees of efficacy and require further clinical validation.

At present, there is no singular intervention universally recognized to mitigate oral or intestinal mucositis that effectively improves the quality of life for cancer patients while also alleviating the burden on healthcare. Pertinently, many current treatments are topical and specific to OM, but mucositis is a condition that can affect the entire GI system. Hence, the identification of a systemic treatment holds significant importance in managing mucositis across the alimentary tract comprehensively. In particular, the development of efficacious preventive interventions for mucositis assumes pivotal significance in optimizing the outcomes of cancer treatment. This emphasis on prevention carries the potential to enhance patient adherence, eliminating the need for treatment interruptions. Consequently, this approach reduces the duration and cost of treatment while simultaneously amplifying treatment efficacy.

For these reasons and given the role of inflammatory mechanisms in the toxic effects of chemotherapy on the alimentary tract, whereby potential therapeutic targets could contribute to mucositis treatment and prevention, our objective in this review was to scrutinise the current evidence base for anti-inflammatory agents that exert a preventive effect on mucositis by undertaking a comprehensive assessment of the literature that included only randomised controlled trials (RCTs).

## 2. Overview of Available Preventative Strategies Using Systemic Anti-Inflammatories for Mucositis

To assess evidence on the use of systemic anti-inflammatory molecules in the prevention of mucositis, a structured search strategy was implemented using the databases PubMed, Ovid, Scopus, and Web of Science, as well as clinical trial registries, including WHO ICTRP and ClinicalTrials.gov. The search was developed by combining the search terms related to four categories, namely (1) the condition, i.e., mucositis (“oral mucositis” was intentionally excluded to include all possible sites); (2) aetiological factors (cancer treatment); (3) the type of medication (anti-inflammatories); and (4) prevention (as opposed to treatment). The following search string was finalised and used to conduct our literature search in April 2023: (mucositis OR “mucosal toxicity” OR “mucosal injury”) AND (prevention OR prophylaxis) AND (“anti-inflammator*” OR NSAID* OR corticosteroid* OR glucocorticoid* OR glucocorticosteroid* OR steroid* or benzydamine) AND (chemotherapy OR radiotherapy OR chemoradiation OR “radiation therapy” OR chemoradiotherapy OR “cancer treatment” OR “targeted treatment” OR “checkpoint inhibitor*”).

While conducting our search, numerous articles were found and excluded that tested the effects of non-systemic administration of anti-inflammatory agents or drugs without direct or clear-cut anti-inflammatory mechanisms. We also excluded studies that investigated the treatment of oral and gastrointestinal mucositis rather than prophylactic methods. The search was updated in April 2024, but no additional relevant publications were identified for this review.

Published RCTs examined interventions with differing outcomes for mucositis prevention [34,35,36,37,38,39,40,41,42,43,44,45,46,47], as summarised in Table 1. Only thalidomide showed a decrease in the incidence of mucositis [34]. Silymarin [35], but not nano-silymarin [36], propolis [37,38], zinc sulphate [39] and thalidomide [34] significantly reduced the severity of mucositis in the intervention group; gabapentin [40] decreased OM symptoms; while melatonin [41] and nanomicelle curcumin [42,43], thalidomide [34], zinc sulphate [39], and silymarin [35,36] use delayed the onset of mucositis. On the other end of the spectrum, studies examining glutamine [44], celecoxib [45], prednisolone [46] and calcitriol [47] did not show any significant difference between the intervention and placebo groups. While not completely preventing CRT-induced mucositis, preventing severe mucositis (grade ≥ 3) and delaying onset can be clinically significant to patients to prevent treatment interruptions and shorten treatment times. Therefore, conducting further investigations into these interventions is crucial for advancing clinical outcomes. An extraction table with detailed description of the main study details is reported in Supplementary Table S1.

## 3. Silymarin

Silymarin, a flavonoid complex derived from *Silybum marianum* (milk thistle) seeds, has attracted substantial scientific interest owing to its notable hepatoprotective, anti-inflammatory, and antioxidant properties [48,49]. The anti-inflammatory effects of silymarin are related to the inhibition of transcription factor nuclear factor-κB (NF-κB) activation in response to TNFα, thereby reducing the expression of pro-inflammatory cytokines and chemokines. This effect is mediated through the suppression of IκB phosphorylation and degradation [50,51].

We identified two RCT studies conducted by the same team that examined the impact of oral silymarin in preventing CRT-induced mucositis. The initial study, by Elyasi et al., evaluated the effectiveness of conventional silymarin tablets, while the subsequent study, conducted by Hosseini et al., aimed to build upon the former by enhancing silymarin bioavailability through a liquid nanoformulation [35,36]. In Elyasi et al.’s study, the median WHO and CTCAE v.3 mucositis scores were significantly lower in the silymarin group at the end of the first to the sixth week (*p* < 0.05). They suggested that conventional oral silymarin tablets might significantly reduce the severity and delay the onset of CRT-induced mucositis in HNC patients [35]. However, Hosseini et al.’s study demonstrated no significant difference in EORTC scores between the nano-silymarin and placebo groups, only observing a non-significant decreasing trend of mucositis severity after four weeks of treatment [36]. Based on the current findings, there is not sufficient evidence to recommend the utilization of this intervention for the prevention of mucositis.

## 4. Glutamine

Glutamine is a vital amino acid found in human blood, skeletal muscle, and the free amino acid pool [52]. A healthy, stable body can produce abundant quantities of this amino acid. However, under metabolic stresses, glutamine becomes limited, increasing the body’s vulnerability to infections and immune responses [53,54]. Studies reveal that glutamine has anti-inflammatory properties and can influence several inflammatory signalling pathways, including the NF-κB and signal transducer and activator of transcription (STAT) pathways [55]. STAT proteins are transcription factors that modulate the immune system, cellular proliferation, and development [56]. They play a vital role in regulating inflammation by inducing the expression of cytokines, including IL-6 [57]. Therefore, glutamine’s anti-inflammatory effect may be attributed to inhibiting STAT activation and the expression of inflammatory cytokines such as IL-6 and IL-8.

In the literature, we found one RCT study that assessed the impact of glutamine in preventing radiation-induced esophagitis among patients with advanced thoracic malignancies [44]. The primary objective was to evaluate the severity of esophagitis, and the results indicated no significant difference in incidence, onset time, or median duration between the two treatment groups. However, this sole study did not yield sufficient evidence to support any recommendation for the use of glutamine in preventing esophagitis resulting from radiotherapy.

## 5. Propolis

Propolis is a resinous substance collected by honeybees from various plant sources. Rich in bioactive compounds such as flavonoids, phenolic acids, and terpenoids, propolis has been investigated for its ability to modulate inflammatory responses [58,59]. Studies have shown that propolis extracts possess antioxidant and anti-inflammatory effects by inhibiting the production of pro-inflammatory cytokines, such as IL-1β, IL-6, and tumour necrosis TNF-α, while promoting the secretion of anti-inflammatory cytokines [60]. The multifaceted nature of propolis’s bioactive components contributes to its potential to mitigate inflammation and support tissue repair. Moreover, propolis’s wound healing properties make it an intriguing candidate for preventing and treating conditions characterized by mucosal injury, such as chemoradiation-induced mucositis [59].

We found two clinical studies that investigated propolis’s preventive and therapeutic effects on cancer therapy-induced mucositis. Bolouri et al. examined the efficacy of water-based extract of propolis mouthwash on RT-induced mucositis of HNC patients, in which the subjects swished and swallowed the solution, providing topical and systemic effects [37]. Salehi et al. evaluated the effect of propolis tablets on CT-induced mucositis in colon cancer patients [38]. These two RCTs examined various formulations of propolis and types of induced mucositis. Across all follow-up assessments, NCI-CTC Mucositis scores were notably lower in the study group, indicating that propolis-based mouthwash is both safe and effective in preventing and treating RT-induced mucositis [37]. In the study involving propolis tablets, based on the WHO Mucositis score, the intervention group exhibited significantly lower average mucositis severity during the second and third follow-up sessions. These findings suggest that systemic propolis tablets have the potential to reduce both the incidence and severity of CT-induced mucositis [38]. Further investigation into the specific mechanisms underlying propolis’s anti-inflammatory actions could provide valuable insights into its potential as a systemic agent for mucositis prevention in cancer patients undergoing treatment.

## 6. Cyclooxygenase-2 (COX-2) Inhibitors (Celecoxib)

Celecoxib, an NSAID, inhibits cyclooxygenase-2 (COX-2), a crucial enzyme in the inflammatory process that triggers heightened production of proinflammatory agents. Tissue injury and pain are mediated by prostaglandin E2 (PGE2) and prostacyclin (PGI2). Pain scores associated with mucositis in patients undergoing high-dose chemotherapy showed correlation with tissue levels of COX-1 and PGE synthase, as well as salivary prostaglandins [61]. Similarly, oral mucosa exhibited a significant increase in tissue levels of NFκB and COX-2 during chemotherapy administration [62]. Therefore, the inhibition of COX-2 could be a useful therapeutic target [45].

A single double-blind RCT evaluated the effects of celecoxib on the prevention of RT-induced OM. Clinical severity of OM was evaluated using the Oral Mucositis Assessment Scale (OMAS), in addition to the severity of mouth pain, normalcy of diet and opioid analgesic use, all showed no significant difference between the intervention and placebo groups [45]. Given the limited available evidence, it is not possible to make a recommendation regarding this intervention.

## 7. Gabapentin

Gabapentin is an antiepileptic drug used to treat neuropathic pain. While its precise mechanisms of action remain unclear, one study suggests that gabapentin’s efficacy in reducing neuropathic pain stems from its interaction with α2δ-1-bound NMDA receptors. In addition to its role as a neuromodulator, gabapentin also exhibits anti-inflammatory properties. Amid concerns regarding the opioid crisis, researchers are actively exploring broader applications of gabapentin, evaluating its potential as an opioid-sparing alternative for various pain conditions. For instance, gabapentin has demonstrated its ability to reduce the necessity for opioid analgesics in perioperative scenarios, where its usage is increasingly common. Multiple studies have investigated gabapentin’s role in mitigating mucositis pain and related symptoms in individuals with head and neck cancer undergoing chemoradiation.

We identified one study that compared standard care (oral health measures, oral rinsing, magic mouthwash, NSAIDs, opioids, and information on oral care and pain management) with prophylactic gabapentin added to standard care for patients undergoing CRT [40]. The study revealed that prophylactic gabapentin led to reduced symptom scores on the Vanderbilt Head and Neck Symptom Survey. Statistically significant decreases were observed in pain (OR: 0.549) and mucosal sensitivity. This study indicated that prophylactic gabapentin, along with standard care, may reduce symptoms associated with OM; however, it may not prevent its development. Although the results are mixed, the data support further investigation of gabapentin in this setting.

## 8. Zinc Sulphate

Zinc, an essential micronutrient, plays a crucial role in various aspects of cellular function, including immune response modulation, cell proliferation, and collagen synthesis, all of which are essential for tissue repair and regeneration [63,64,65]. Through its participation in enzymatic activities and gene expression, zinc contributes to the maintenance of skin and mucosal integrity. Studies have highlighted zinc’s ability to facilitate epithelial cell turnover, enhance fibroblast proliferation, and support the formation of granulation tissue [66]. Zinc’s anti-inflammatory properties have been widely documented, with studies showing its ability to modulate immune responses and inhibit pro-inflammatory cytokines like IL-1β and TNF-α [67,68].

We found one study that examined zinc sulphate as a prophylaxis for OM patients receiving RT [39]. This study examined OM onset timing, the dose of RT at onset, severity, and post-RT OM. This study showed that prophylactic zinc sulphate had a significant effect in delaying OM onset and at increased RT dose, as well as decreasing its severity and greatly decreasing the presence of OM post-RT treatment. This study concluded that zinc sulphate could decrease OM severity and improve healing of OM-related lesions. Investigating zinc’s potential preventive effects on chemoradiation-induced mucositis warrants further attention.

## 9. Corticosteroids (Prednisone)

Corticosteroids, synthetic hormones resembling those naturally produced by the adrenal glands, are renowned for their potent anti-inflammatory effects. They have been explored as potential interventions for various inflammatory conditions [69,70]. These compounds exert their actions by modulating immune responses, suppressing cytokine production, and inhibiting the activation of immune cells, while enhancing the production of anti-inflammatory proteins [70,71,72]. This dual mechanism effectively suppresses immune responses and mitigates inflammation, rendering corticosteroids valuable in treating diverse inflammatory conditions, including mucositis.

We identified one study examining the effect of prednisone on mucositis and mucositis-related complications during RT and reported a significant decrease in RT treatment time with prednisone use [46]. No significant difference was found in the degree or duration of mucositis between treatment and placebo groups or in treatment interruption beyond 3 days. This single study provides insufficient evidence regarding the efficacy of corticosteroids in the prevention of RT-induced mucositis. Further research is needed to clarify their potential and optimize their usage in mucositis prevention.

## 10. Curcumin

Curcumin, a bioactive compound derived from the turmeric plant, has gained recognition for its potent anti-inflammatory properties [73]. Its molecular mechanisms involve inhibiting various signalling pathways that drive inflammation, such as NF-κB and mitogen-activated protein kinases (MAPKs). Curcumin’s ability to downregulate the expression of pro-inflammatory cytokines, chemokines, and enzymes involved in inflammation underscores its potential as a multifaceted anti-inflammatory agent. The modulation of these pathways by curcumin not only attenuates the inflammatory response but also supports tissue repair and regeneration processes. Given its well-documented anti-inflammatory effects, curcumin may offer promise in preventing mucositis induced by cancer treatments. By targeting the underlying inflammatory mechanisms that contribute to mucosal damage, curcumin could serve as a preventive strategy to alleviate the severity and onset of mucositis. However, further research is essential to validate its efficacy, optimal dosing, and potential interactions with other therapies.

Two RCTs investigated the effectiveness of oral nanomicelle curcumin in managing CT- and/or RT-induced OM. Delavarian et al. focused on RT-induced OM in HNC patients [42]. Their trial assessed OM severity and occurrence using the NCI-CTC v.2 scale, revealing a statistically significant delay in OM onset and lower mucositis grade in the study group [42]. Kia et al. showed a significantly lower severity of OM in the study group in weeks 1, 4, and 7, but not in week 2. Also, the mean pain score was significantly lower in the study group in week 7 [43]. Notably, there was significantly lower OM severity in patients undergoing CT alone in the study group in all weeks assessed. In contrast, study group patients undergoing CRT showed lower OM severity only in weeks 4 and 7 [43]. Although the mean pain score consistently differed significantly between groups for patients undergoing CT (*p* < 0.001), it did not show statistical significance for patients undergoing CRT [43].

Given its well-documented anti-inflammatory effects, curcumin may offer promise in preventing mucositis induced by cancer treatments. By targeting the underlying inflammatory mechanisms that contribute to mucosal damage, curcumin could serve as a preventive strategy to alleviate the severity and onset of mucositis. However, further research is essential to validate its efficacy, optimal dosing, and potential interactions with other therapies.

## 11. Melatonin

Melatonin is an indoleamine derived from tryptophan and synthesized in the pineal gland as well as other tissues [74,75]. Extensive research highlights melatonin’s ability to modulate inflammation and oxidative stress, key factors in the pathogenesis of various diseases [74,76]. Through its antioxidant properties, melatonin scavenges free radicals and mitigates oxidative stress-induced damage. Furthermore, melatonin exerts anti-inflammatory effects by suppressing the production of pro-inflammatory cytokines, inhibiting NF-κB activation, and regulating immune responses. These multifaceted actions position melatonin as a potential agent for preventing mucositis induced by cancer treatments [76].

We identified one study which examined the effect of melatonin in HNC patients undergoing CRT [41]. The test group reported decreased grade 3 mucositis and grade 2 xerostomia; however, this was not statistically significant. There was a significant delay in the onset of grade 3 mucositis vs. the placebo group and a significant decrease in morphine consumption. This study concluded that whilst melatonin does not prevent mucositis, it may delay onset and symptoms and allow for fewer treatment interruptions. This single study provides insufficient evidence regarding the efficacy of corticosteroids in the prevention of CRT-induced mucositis. However, further investigations are needed to validate its efficacy.

## 12. Thalidomide

Initially developed as a sedative and anti-nausea medication, thalidomide gained notoriety due to causing severe birth defects in pregnant women, leading to its market withdrawal. Despite its history as a human teratogen, thalidomide is emerging as a treatment for cancer and inflammatory diseases [77]. Its potent anti-inflammatory effects stem from its ability to inhibit TNF-α and suppress NF-κB activation in response to inflammatory agents. Thalidomide has demonstrated its efficacy in several diseases, including various inflammatory conditions like rheumatoid arthritis, Crohn’s disease, and Behcet’s disease. Moreover, thalidomide is linked to a range of immunomodulatory actions [78].

One study by Liang et al. examined the effects of thalidomide on mucositis latency, incidence, and severity in patients with nasopharyngeal carcinoma undergoing concurrent CT [34]. In the test group, the OM latency period was increased significantly. Additionally, the incidence of both OM and severe OM (grade 3 or higher) was significantly reduced in comparison to the control, indicating that thalidomide may help to prevent OM in nasopharyngeal carcinoma patients.

## 13. Calcitriol

Calcitriol, the active form of vitamin D, has been recognized not only for its crucial role in calcium homeostasis and bone health but also for its potential anti-inflammatory properties [79,80]. Vitamin D, as a pleiotropic compound, plays a fundamental role in immunoregulation through its receptors expressed in diverse myeloid and lymphoid cells [81]. Experimental investigations have demonstrated that vitamin D is capable of reducing the release of TNF-α and increasing the synthesis of the anti-inflammatory cytokine IL-10 [82]. Additionally, vitamin D has been shown to induce the synthesis of antimicrobial peptides, such as defensin and cathelicidin, in immune cells [83]. Moreover, animal studies highlight vitamin D’s anti-inflammatory effects in conditions like inflammatory bowel disease, where it can modulate cytokines such as IL-1β, IL-10, and IL-17 [84]. Vitamin D supplementation in patients with leg ulcers has been suggested to promote better lesion healing, possibly by inducing the synthesis of platelet-derived growth factor, a key growth factor in wound healing, and subsequently stimulating collagen production by fibroblasts [85]. Studies have also elucidated vitamin D’s antioxidant activity, demonstrating its ability to combat oxidative stress through the upregulation of glutathione peroxidase and superoxide dismutase, as well as by modulating free radical formation [86].

We found one study which examined the effect of calcitriol on OM incidence and severity in Fanconi anaemia patients undergoing high dose CT in preparation of HSCT [28,47]. Despite focusing on anaemic patients, we included this study due to the similarity in the use of high-dose CT, known to induce mucositis, in cancer patients. All participants received prophylaxis conditioning regimens consisting of nystatin drops, sucralfate, and diluted povidone-iodine. While the study group received calcitriol, the results indicated no significant difference in OM incidence or severity compared to the placebo group. However, this study did reveal a significant association between baseline serum vitamin D levels and OM resolution. Considering the limitation of available evidence, it is not feasible to provide a recommendation regarding this intervention.

## 14. Limitations of Current Evidence

The studies included in this review had various limitations or methodological inadequacies. Liang’s study on thalidomide was open label with no placebo in the control group. As such, it may have been subject to measurement bias from the researchers and the psychosomatic differences in patients not controlled for [34]. Additionally, the study lacked information on patients’ nutritional supplementation and analgesic use, which may confound the results. The studies on melatonin and propolis involved treatments with oral liquids that potentially affected OM sites both topically and systemically [38,41]. Analysing each agent separately via different administration routes could provide clarity on their effects and illuminate the therapeutic mechanisms involved in mucositis prevention.

Another important limitation of the study lies in comparing the effects of systemic anti-inflammatory drugs across different treatment modalities, such as radiotherapy and chemotherapy, which may lead to a potential bias. The local physical effects of radiotherapy and systemic chemical effects of chemotherapy present challenges in evaluating the efficacy of systemic anti-inflammatory drugs uniformly. Future research should address these distinctions for a clearer understanding of mucositis prevention and treatment. Many of the included studies evaluated the interventions with CT, RT, or CRT. Each agent should be tested with separate treatment populations to best evaluate how the effects may differ.

Several limitations shared by many of the studies indicated the crucial need for future research to expand knowledge about the potential interventions for mucositis prevention. In Hosseini et al.’s study on silymarin, the timing of the intervention relative to the start of cancer treatment may be necessary to the mucositis outcomes [46]. Sufficient serum levels of systemic medications will be necessary to have a measurable clinical effect on the development of mucositis. The effect of taking these agents at different time points should be evaluated. Relevant to timing is the dosages of agents. As there is limited research on this topic, with often only one study for each of these agents, further research is required to evaluate different dosages to determine whether an outcome is indeed dose-related and effect size can be increased for improved patient outcomes. Furthermore, patients should undergo blood tests to determine if there are detectable serum levels of the agents and at what levels they are associated with study outcomes. Furthermore, specifically evaluating the mechanisms by which anti-inflammatory agents interrupt CRT-induced mucositis pathway could indicate further routes to follow for mucositis prevention and which agents may be more effective.

Another shared limitation across many other studies was a small sample size limiting the power of the studies [35,37,38,39,42,43,45,46,47]. Given the small sample sizes in the included studies, caution should be exercised when generalising the findings. Future research should include larger, more diverse samples, while employing multicentre studies or longitudinal designs may provide more robust and generalisable findings. This is especially true with cancer patients taking multiple medications that may interact with treatments differently.

Varying mucositis scoring systems among studies may create data diversity due to differing criteria, grading scales, and severity definitions. These scales may focus on different aspects like clinical features, functional outcomes, or symptoms, making direct comparison and result synthesis difficult, impacting literature review conclusions. Efforts to standardise mucositis scoring systems and establish consensus guidelines would help to improve the comparability and reliability of future studies [87].

We focused on systemic agents, as these have the potential to be deposited throughout the entire GI tract through the oral route and exert their effects, reaching areas that may not be accessible to non-systemic agents [88]. Systemic administration through the parenteral route was also considered, as it ensures the consistent and controlled delivery of the agent, ensuring that therapeutic levels are reached and maintained in the affected tissues [89].

## 15. Conclusions

This review revealed that in the literature, several anti-inflammatory agents have been tested with the aim of preventing mucositis in cancer patients undergoing CT and/or RT. From the RCTs evaluated in this study, only a few compounds appear to show potential efficacy in being mucositis-preventive, and of those, most were only tested in one study. Notably, many of the studies suffer from small sample sizes, raising concerns regarding their statistical power. In all, the current evidence in the literature in relation to systemic anti-inflammatories appears limited at best, and further research is warranted to yield more extensive results.

## Figures and Tables

**Table 1:** Summary of the main features of the clinical studies included.

Author, Year	Study Type (Time)	Population	Intervention	Comparator	Outcomes	Summary of Main Effect Observed
Liang et al., 2022 [34]	Multicentre RCT (5 months)	Adults (*N* = 155) with nasopharyngeal carcinoma undergoing CRT	Thalidomide (75 mg) + basic oral hygiene guidance (*N* = 76)	Basic oral hygiene guidance (*N* = 79)	OM severity (WHO); mouth and throat soreness; body weight; adverse events	Intervention group had a significantly longer latency period and lower incidence of OM compared to control
Elyasi et al., 2016 [35]	Prospective double-blind RCT (6 weeks)	Adults (N = 29) with HNC undergoing CRT	Conventional silymarin tablets (140 mg) (*N* = 13)	Placebo tablets (*N* = 14)	OM severity (WHO and CTCAE v.3)	Significantly lower OM severity and intolerable mucositis (stage 3–4) in the intervention group compared to control
Hosseini et al., 2021 [36]	Double-blind RCT (6 weeks)	Adult SCC patients (N = 31) undergoing CRT	Nano-silymarin solution (70 mg/5 mL) (*N* = 16)	Placebo solution (5 mL) (*N* = 15)	OM severity (RTOG)	The intervention group had a non-significant decreasing OM severity trend compared to controls
Bolouri et al., 2015 [37]	Triple-blind RCT (5 weeks)	HNC patients >15 years old undergoing RT *(N* = 20)	Propolis mouthwash (3%, 15 mL, swish and swallow) (*N* = 10)	Placebo mouthwash (15 mL, swish and swallow) (*N* = 10)	OM severity (NCI-CTC v.2); body weight	OM severity and mean weight loss in intervention group was significantly lower than in controls
Salehi et al., 2018 [38]	Double- blind RCT (3 weeks)	Adults (*N* = 50) with colon cancer undergoing CT	Propolis (50 mg) capsule (*N* = 25)	Placebo capsule (*N* = 25)	OM severity (WHO)	There was a significant decrease in OM severity with intervention compared to control at day 14 and 21
Ertekin et al., 2004 [39]	Prospective double-blind RCT (13 weeks)	Adults (*N* = 27) with HNC undergoing RT only, or RT with concurrent CT	Zinc sulphate (50 mg zinc) capsule *(N* = 15)	Placebo capsule (*N* = 12)	OM severity (RTOG); body weight	OM onset was delayed in the intervention group and occurred at a lower severity and at an increased RT dose compared to controls
Smith et al., 2020 [40]	Prospective RCT, no blinding (9 months)	Adults (*N* = 71) with HNC undergoing CRT	Standard therapy * + gabapentin capsule (*N* = 39)	Standard therapy. * (N = 32)	OM pain score (VHNSSv2); General Symptom Survey	OM pain and other symptoms were significantly reduced in the intervention group compared to controls
Onseng et al., 2017 [41]	Double-blind RCT (7 weeks)	Adults (*N* = 39) with HNC undergoing CRT	Melatonin solution (10 mL, 0.2%) + melatonin capsule (20 mg) (*N* = 19)	Placebo solution (10 mL) + Placebo capsule (*N* = 20)	OM severity (WHO); xerostomia (CTCAE v4.03); Quality of Life scores (FACT-H&N); OM pain score (VAS)	Grade 3 OM onset was delayed by a median of 16 days and median morphine consumption for pain control was significantly lower in the intervention group compared to control
Delavarian et al., 2019 [42]	Double-blind RCT (6 weeks)	Adults (*N* = 29) with HNC undergoing RT	Nano-curcumin (80 mg) soft gel (*N* = 15)	Placebo capsule (*N* = 14)	OM severity (NCI-CTC v.2); body weight	Delay in onset and reduced severity of OM, and reduced body weight loss were observed in the intervention group
Kia et al., 2021 [43]	Double-blind RCT (7 weeks)	Cancer patients (*N* = 50) undergoing CT only, or CRT	Nano-curcumin (80 mg) soft gel (*N* = 25)	Placebo capsule (*N* = 25)	OM severity (WHO); OM pain score (NRS)	OM severity was significantly lower both for patients undergoing CT (all weeks) and for those undergoing CRT (weeks 4 and 7), with mean pain score significantly lower, in the intervention group compared to controls
Alshawa et al., 2021 [44]	Double-blind RCT (9 months)	Adults (*N* = 38) with thoracic malignancies undergoing RT or CRT	Glutamine suspension (*N* = 19)	Placebo (glycine) suspension (*N* = 19)	Esophagitis severity (CTCAE v4.03); body weight; symptom burden (MDASI-HN); Study Medication Satisfaction Scale	No significant differences observed for outcome except core symptom severity, which was higher in the intervention group
Lalla et al., 2014 [45]	Prospective multicentre, double-blind RCT (8 weeks)	Adults (*N* = 40) with HNC undergoing RT	Celecoxib (200 mg) capsule (*N* = 19)	Placebo capsule (*N* = 20)	OM severity (OMAS, WHO, CNI-CTCv.2); pain score (Brief Pain Inventory); analgesic use; diet (Performance Status Scale)	No mean OM severity (on all scales used), pain scores, normalcy of diet, nor opioid analgesic use had a significant difference between groups
Leborgne et al., 1998 [46]	Double-blind RCT (13 weeks)	SCC patients (*N* = 66) undergoing RT	Prednisone capsules (20–40 mg) (*N* = 32)	Placebo capsule (*N* = 34)	Total duration of treatment and interruptions; OM severity (WHO); hospitalisation and nutritional support; body weight	Significant decrease in RT treatment time for the intervention group; body weight loss average was less severe in the intervention group
Hamidieh et al., 2016 [47]	Double-blind RCT (5 weeks)	Children (*N* = 28) with Fanconi anaemia receiving high-dose CT conditioning regimen prior to undergoing allogeneic HSCT.	OM prophylaxis regimen ** + Calcitriol (0.025 μg) capsule (*N* = 14)	OM prophylaxis regimen * + Placebo capsule *(N* = 14)	OM severity (WHO); baseline serum 25-OH vitamin D level	Baseline sufficient (>20 ng/mL) vitamin D level was significantly associated with complete OM resolution to grades 0–1; recovery of grades 3–4 OM to lower grades was significantly associated with non-deficient vitamin D levels.

* Standard Supportive care included brushing with fluoride toothpaste; flossing; oral rinsing with baking soda and salt water every 2–3 h; “miracle mouthwash” containing topical lidocaine, diphenhydramine, and aluminium magnesium hydroxide; nonsteroidal anti-inflammatories around the clock; opioid analgesics as needed. ** Hospital protocol for OM prophylaxis included nystatin 15–20 drops every 3 h, sucralfate 500 mg chewable tablet every 6 h, and 10 mL diluted povidone-iodine every 3 h. Abbreviations. CT: chemotherapy; CRT: chemoradiotherapy; HNC: head and neck cancer; HSCT: haematopoietic stem-cell transplantation; NCI-CTC v.2: National Cancer Institute Common Toxicity Criteria version 2, severity grading scale for radiation-related mucositis CTCAE v4.03: Common Toxicity Criteria for Adverse Events scale, version 4.03; FACT-H&N: Functional Assessment of Cancer Therapy—Head and Neck Version 4; MDASI-HN (MD Anderson Symptom Inventory Head and Neck module; NRS: eleven-point Numerical Rating Scale; OMAS: Oral Mucositis Assessment Scale; RCT: randomised controlled trials; RT: radiotherapy; RTOG: acute radiation morbidity mucosal scoring criteria from the Radiation Therapy Oncology Group and the European Organization for Research and Treatment of Cancer (EORTC); SCC: squamous cell carcinoma; WHO: World Health Organization severity grading scale for oral mucositis; VAS: Visual Analogue Scale for pain assessment; VHNSSv2: Vanderbilt Head and Neck Symptom Survey, version 2.

## Data Availability

Data is contained within the article or Supplementary Materials.

## Supplementary Materials

The following supporting information can be downloaded at: https://www.mdpi.com/article/10.3390/biom14050560/s1, Table S1: Extraction table with summary of clinical results.
