# Supplementary material for: Systemic Anti-Inflammatory Agents in the Prevention of Chemoradiation-Induced Mucositis: A Review of Randomised Controlled Trials
Source: Biomolecules. 2024 May 6;14(5):560. doi: 10.3390/biom14050560 (PMC11117894; doi:10.3390/biom14050560)
Supplement: Supplementary file 1 [file biomolecules-14-00560-s001.zip › biomolecules-2937831-supplementary.pdf]

Systemic Anti-Inflammatory Agents in the Prevention of Chemoradiation-Induced Mucositis: A Review of Randomised Controlled Trials

Supplementary Table S1: Extraction table with summary of clinical results

AUTHOR, YEAR	STUDY TYPE (TIME FOLLOWING EACH SUBJECT)	POPULATION	INTERVENTION (STUDY GROUP)	COMPARATOR (CONTROL GROUP)	OUTCOME, UNITS OF MEASUREMENT, ASSESSMENT INTERVALS	EFFECT OBSERVED
Liang et al., 2021 (35)	Multicenter RCT (5 months)	Adults with nasopharyngeal carcinoma undergoing CRT (50-74 Gy in 30-33 fractions, 5 times a week; 100 mg/m^2 IV Cisplatin on days 1, 22, and 43). *N* = 155	Thalidomide (75 mg) every night orally) + Basic oral hygiene guidance and normal saline gargle (15 mL) tds, after meals. *N* = 76	Basic oral hygiene guidance and normal saline gargle (15 mL) tds, after meals. *N* = 79	-OM severity (WHO) recorded daily. -Mouth and throat soreness recorded daily. -Body weight measured before and after treatment. -Adverse events (CTCAE v4.03) monitored during and up to 3 months after CRT.	-Significantly prolonged latency period of OM in S (30 days vs. 14 days in C, P <0.001). -S had a significantly lower incidence of OM (87.5% vs 97.5%, P=0.016) and severe OM (27.5% vs 46.3%, P=0.014) compared to C. -Mouth and throat soreness score was significantly lower in S (P=0.026). -Lower mean weight loss in S (4.3 ± 1.7 kg) compared to C (5.6 ± 2.5) (*P* < .001). -Two patients were excluded from the study as they had unbearable dizziness after taking THD. The incidence of dizziness and constipation were significantly higher in S, while the incidence of nausea, vomiting, and insomnia were significantly lower in S.
Elyasi et al., 2016 (36)	Prospective double- blind RCT (6 weeks)	Adults with HNC undergoing CRT (50-70 Gy, exposed to ≥50% of the oral cavity; 100 mg/m^2 cisplatin on days 1, 22, and 43). *N* = 29	Conventional silymarin tablets (140mg), tds, after meals. *N* = 13	Placebo tablets, tds, after meals. *N* = 14	-OM severity (WHO and CTCAE v.3) assessed weekly from the beginning of RT.	-The median OM severity (both scales) was significantly lower in S compared to C at the end of each week (P<0.05). -Intolerable mucositis (stage 3-4) occurrence between the groups at the end of the first 3 weeks was not significantly different; however, incidence was significantly higher in C at the end of week 4 was noted (based on NCI-CTC scale, P=0.004) and at the end of weeks 5 and 6 of (both scales, P<0.001). -OM severity was significantly different between week 1 and 2 for C, but not for S, which may indicate a delay in onset. -No adverse effects related to the study drug were reported.

Hosseini et al., 2021 (37)	Double- blind RCT (6 weeks)	Adult SCC patients undergoing CRT (30-33 fractions of 1.8-2 Gy each, 5 days per week; 40 mg/m^2 weekly Cisplatin). *N* = 31	Nano-silymarin solution (70mg/5mL), tds, with meals. *N* = 16	Placebo solution (5mL), tds, with meals. *N* = 15	-OM severity (RTOG) assessed weekly for 6 weeks from the first day of CRT.	-OM scores increased significantly in both groups during RT but were not significantly different between the two groups anytime during the study (P=0.157, both groups had a median score of 3 at the end of the study period). -After 4 weeks of treatment with silymarin, the S had a non-significant decreasing OM severity trend compared to C. -80% of patients complained of unpleasant taste. Nausea or abdominal pain was experienced by 5 patients, although these adverse reactions are indistinguishable from those which may be experienced from CT.
Bolouri et al., 2015 (38)	Triple- blind RCT (5 weeks)	HNC patients >15 years old undergoing RT (50-70 cGy). *N* = 20	Propolis mouthwash (3%, 15mL), tds, swish and swallow. *N* = 10	Placebo mouthwash (15mL), tds, swish and swallow. *N* = 10	-OM severity (NCI-CTC v.2) evaluated weekly, from beginning to end of RT. -Body weight measured at start and end of treatment.	-OM severity in S was significantly lower than in C at each follow-up. (P<0.05) -8/10 S patients did not develop any mucositis during RT. -Mean weight loss in S was significantly lower than in C (0.2 kg vs 3.4 kg, P=0.029).
Salehi et al., 2018 (39)	Double- blind RCT (3 weeks)	Adults with colon cancer undergoing CT (Folfox Diet: Oxaliplatin, Lacorin, Fluoracillin) *N* = 50	Propolis (50 mg) capsule, bd, with food. *N* = 25	Placebo capsule, bd, with food. *N* = 25	-OM severity (WHO) evaluated prior to CT and at the end of weeks 1, 2, and 3.	-OM severity average was not significant at day 7 (0.98 for S vs 1.15 for C, P=0.312). -There was a significant decrease in OM severity in S compared to C at day 14 (0.8 vs 1.4, P=0.027) and 21 (0.52 vs 1.00, P=0.039).
Ertekin et al., 2004 (40)	Prospective double- blind RCT (13 weeks)	Adults with HNC undergoing RT only (≥4000 cGy in 2 Gy fractions 5 times a week, within 4-7 weeks; *N* = 21), or RT with concurrent CT (*N* = 6). *N* = 27	Zinc sulphate (50 mg zinc) capsule, tds, from start of RT until 6 weeks after completion. *N* = 15	Placebo capsule, tds. *N* = 12	-OM severity (RTOG) evaluated prior to RT, weekly during RT, the first day after completion, and 6 weeks after treatment. -Body weight measured at the same intervals.	-OM onset was delayed in S (P<0.05), appearing in week 3 compared to onset in week 2 for C. -OM onset occurred at an increased RT dose in S (36Gy vs 20Gy for C, P<0.01). -OM occurred at a lower severity in S, on average reaching Grade 1 compared to Grade 3 in C (P<0.05). -At 6 weeks post-RT, OM was found in significantly fewer S patients (1/15 vs 10/12 in C), P<0.01). However, RTOG grade 3 nausea and vomiting developed in 3 S patients. -Weight loss increased as RT advanced in both groups (P<0.001), but the difference between groups was not significant.

Smith et al., 2020 (41)	Prospective RCT, no blinding (9 months)	Adults with HNC undergoing CRT (weekly carboplatin and taxol, or single agent cisplatin weekly or every 3 weeks; 50-70 Gy). N = 71	Standard therapy* + Gabapentin capsule, taken in escalating doses, tds: 100 mg in week 1, 300 mg in week 2, 600 mg in week 4, 900 mg from week 4 onward. *N* = 39	Standard therapy. * *N* = 32	- OM pain score (VHNSSv2) assessed at baseline, weekly during therapy, and at 1-, 2-, and 3-months post-CRT. -General Symptom Survey completed at the same time points, to evaluate systemic symptoms.	-OM pain was significantly reduced (>1.5/10 points) in S (OR = 0.549, 95% CI:[0.364, 0.827],P= .004). -Decreases in symptom scores were observed in S for: chemosensory symptoms (taste and smell) P= .029, mucosal sensitivity (P= .007), and xerostomia (P= .010). -Systemic symptoms were significantly decreased in S, including unexplained fatigue and sweating, problems staying asleep, and feeling sad, depressed, or anxious (P< .001). -No severe adverse reactions were reported;however, toxicities preventing dose escalation and in some cases leading to discontinuation of the drug included fatigue, drowsiness, and sedation.<br><br>*Supportive care included brushing with fluoride toothpaste; flossing; oral rinsing with baking soda and salt water every 2-3 hours; “miracle mouthwash” containing topical lidocaine, diphenhydramine, and aluminum magnesium hydroxide; nonsteroidal anti-inflammatories around the clock; opioid analgesics as needed.
Onseng et al., 2017 (42)	Double- blind RCT (7 weeks)	Adults with HNC undergoing CRT (5 days per week with a planned dose of ≥50Gy; Cisplatin 20-40 mg/m^2 weekly for 6 cycles or 100 mg/m^2 every 3 weeks for 6 cycles. *N* = 39	Melatonin solution (10 mL, 0.2%), gargle for 2-5 minutes then swallow within 15 minutes prior to RT + Melatonin capsule (20 mg) daily before bedtime. *N* = 19	Placebo solution (10 mL), gargle for 2-5 minutes then swallow within 15 minutes prior to RT + Placebo capsule, taken daily before bedtime. *N* = 20	-OM severity (WHO) assessed at baseline (day 0) and daily until the end of RT (day 49). -Xerostomia (CTCAE v4.03) measured daily. -Quality of Life scores (FACT-H&N) measured at baseline, and weeks 3 and 7. -OM pain score (VAS) measured daily, then oral morphine equivalent amount used for analgesic treatment was recorded as prescribed.	-Grade 3 OM was reported in a lower proportion of S compared to C, but no statistical significance was detected. -Grade 2 xerostomia was reported in approximately the same proportion of each group (20% in S vs 21% in C), and there was no difference in time until onset (median 50 visits in both groups). -Grade 3 OM onset was delayed by a median of 16 days in S (P=0.0318). One S patient experienced treatment interruptions compared to five C patients. -Median morphine consumption for pain control was significantly lower in S (0 mg vs 57 mg in C; P=0.034). -Quality of life scores decreased for both groups during treatment, but there was no significant difference between groups. -No serious adverse events related to the drug were reported.
Delavarian et al., 2019 (43)	Double- blind RCT (6 weeks)	Adults with HNC undergoing RT (≥50 Gy, exposed to ≥50% of the oral cavity). *N* = 29	Nano-curcumin (80 mg) soft gel, daily. *N* = 15	Placebo capsule, daily. *N* = 14	-OM severity (NCI-CTC v.2) evaluated weekly from the start of RT (day 0) until completion (day 42). -Body weight measured before and after treatment.	-S experienced a delay in onset of OM compared to C (grade 1 mucositis exhibited in 0% vs 37.5% of cases in week 1, 25% vs 50% of cases in week 2, P=0.002). -OM severity increased in all patients and was greatest after 6 weeks but was significantly lower in S (P<0.05) in all weeks. No patient in S developed grade 4 mucositis by the end of RT. -Body weight loss average was used to assess nutritional condition, was significantly lower in S

						(0.43 +/- 0.81 kg study vs 1.32 +/- 0.87 control, P=0.003)<br>-No side effects or discomfort were found from the drug.
Kia et al., 2021 (44)	Double- blind RCT (7 weeks)	Cancer patients undergoing CT only (Cisplatin 30-50 mg and 5FU 640–750 mg; *N* = 37), or CRT (CT + 6000–7000 cGy; *N* = 13).<br>*N* = 50	Nano-curcumin (80mg) soft gel, bd, after food.<br>*N* = 25	Placebo capsule, bd, after food.<br>*N* = 25	-OM severity (WHO) evaluated at week 1, 2, 4, and 7.<br>-OM pain score (NRS) evaluated at week 1, 2, 4, and 7.	-Mean OM severity was significantly higher in C compared to S in weeks 1 (0.72 vs 0.36, P=0.010), 4 (1.88 vs 1.44, P=0.022), and 7 (2.2 vs 1.36, P<0.001).<br>-Mean OM severity significantly gradually increased over 7 weeks in C (P<0.001) and up to 4 weeks for S (P<0.001) while insignificantly decreasing from weeks 4-7 for S.<br>-OM severity was significantly lower for S patients undergoing CT in all weeks (P<0.001) compared to C, while it was significantly lower in S for patients undergoing CRT in weeks 4 (P=0.009) and 7 (P=0.012).<br>-Mean pain score significantly increased throughout the study in both groups (P<0.001); however, it was significantly lower in S compared to C in week 7 (2.64 vs 4.44, P=0.001).<br>-The difference in mean pain score between groups was significant over the length of the study for patients undergoing CT only (P<0.001), but not significant for patients undergoing CRT (P=0.128).
Alshawa et al., 2021 (45)	Double- blind RCT (9 months)	Adults with thoracic malignancies undergoing RT (≥45 Gy) or CRT.<br>*N* = 38	Glutamine suspension (4 g mixed with water), bd, initiated between day 1 and 10 of RT, continued for 4 weeks after the completion.<br>*N* = 19	Placebo (glycine) suspension (4 g mixed with water), bd, initiated between day 1 and 10 of RT, continued for 4 weeks after the completion.<br>*N* = 19	-Esophagitis severity (CTCAE v4.03) assessed at baseline, weekly during RT, and at 1 and 6 months after completion.<br>-Body weight measured at the same intervals.<br>-Symptom burden assessments (MDASI-HN) assessed at baseline, weeks 3, 5, 7, at the end of RT, and 1 and 6 months after completion.<br>-Study Medication Satisfaction Scale administered at the end of RT.	-Incidence of esophagitis (any grade or grade 3) in the first 6 weeks of RT had no significant difference between groups (P=1.00, P=0.73, consecutively).<br>-Median time to develop esophagitis had no significant difference between groups (3.4 weeks for S vs 4.1 weeks for C, P=0.29).<br>-Median duration of esophagitis was not significantly different between groups (P=0.54).<br>-S had significantly higher core symptom severity compared to C (2.1 vs 1.5, P < 0.03). No significant differences were found in head and neck specific symptom severity (P<0 .60) or in symptom interference (P<0.22) between groups.<br>-Groups had no significant differences in study medication satisfaction.<br>-Median weight loss was greater in C, but not significantly different from S (2.8 kg vs 0.9 kg, P=0.83).<br>-Five patients in S had grade 1 or 2 adverse events possibly related to the study drug. One grade 2 adverse event was reported in C.
Lalla et al., 2014 (46)	Prospective multicenter, double- blind RCT (8 weeks)	Adults with HNC undergoing RT (≥5000 cGy to ≥2 of 14 pre-defined oral sites).<br>*N* = 40	Celecoxib (200 mg) capsule, bd, from 5 days before the start of RT until 3 days after completion.<br>*N* = 19	Placebo capsule, bd, from 5 days before the start of RT until 3 days after completion.<br>*N* = 20	-OM severity (OMAS, WHO, and CNI-CTC v.2) assessed 2-3 times per week.<br>-OM pain score (Brief Pain Inventory) assessed 2-3 times per week.	-Mean OM severity (on all scales used), pain scores, normalcy of diet, nor opioid analgesic use in IV morphine equivalents had a significant difference between groups, in either an intention-to-treat analysis (all data included regardless of study drug compliance status) or per-protocol analysis

					-Normalcy of diet assessed using Performance Status Scale for HNC patients.<br>-Analgesic use, collected from daily diary and assessed using the Advanced Opioid Converter.	(assessment data included through last compliance date).<br>-No adverse events were detected in S.
Leborgne et al., 1998 (47)	Double- blind RCT<br>(13 weeks)	SCC patients undergoing RT (1.6Gy, bd, for 5 days per week; aiming for 64-65 Gy in 26-29 days).<br>*N* = 66	Prednisone capsules (40 mg from day 8-29 of treatment; 20 mg from day 29-33; 20 mg every other day from day 34-43).<br>*N* = 32	Placebo capsule, once daily.<br>*N* = 34	-Total duration of treatment and interruptions.<br>-OM severity (WHO) evaluated twice weekly during RT and once weekly thereafter up to day 90.<br>-Need for hospitalisation, parenteral or nasogastric tube nutritional support.<br>-Body weight measured before and after treatment.	-Significant decrease in RT treatment time for S (29.9 S v 34.3 C, P=0.013).<br>-Body weight loss average was less severe in S (6% vs 8%, P=0.02).<br>-No significant difference with degree or duration of mucositis expression, interruptions in treatment longer than 3 days, hospitalizations, or nutritional support between groups.<br>-No toxicity to prednisone was observed.
Hamidieh et al., 2016<br>(48)	Double- blind RCT<br>(5 weeks)	Children with Fanconi anaemia receiving high-dose CT conditioning regimen prior to undergoing allogeneic HSCT.<br>*N* = 28	OM prophylaxis regimen* + Calcitriol (0.025 µg) capsule, daily.<br>*N* = 14	OM prophylaxis regimen* + Placebo capsule, daily.<br>*N* = 14	-OM severity (WHO) evaluated daily from the first day of CT until 21 days after transplantation or until resolution of OM.<br>-Baseline serum 25-OH vitamin D level.	-Calcitriol had no significant impact on OM incidence (P=1.00) all but one patient in each group developed OM) or severity (P=0.54).<br>-Baseline sufficient (>20 ng/mL) vitamin D level was significantly associated with complete OM resolution to grades 0-1 (P=0.03), and recovery of grades 3-4 OM to lower grades was significantly associated with non-deficient vitamin D levels (P=0.04).<br><br>*Hospital protocol for OM prophylaxis included nystatin 15-20 drops every 3 hours, sucralfate 500mg chewable tablet every 6 hours, and 10mL diluted povidone-iodine every 3 hours.

Abbreviations. bd: twice a day; C: subjects in the control group; CT: chemotherapy; CRT: chemoradiotherapy; HNC: head and neck cancer; HSCT: haematopoietic stem-cell transplantation; NCI-CTC v.2: National Cancer Institute Common Toxicity Criteria version 2, severity grading scale for radiation-related mucositis CTCAE v4.03: Common Toxicity Criteria for Adverse Events scale, version 4.03; FACT-H&N: Functional Assessment of Cancer Therapy - Head and Neck Version 4; MDASI-HN (MD Anderson Symptom Inventory Head and Neck module; NRS: eleven-point Numerical Rating Scale; OMAS: Oral Mucositis Assessment Scale; RCT: randomised controlled trials; RT: radiotherapy; RTOG: acute radiation morbidity mucosal scoring criteria from the Radiation Therapy Oncology Group and the European Organization for Research and Treatment of Cancer (EORTC); S: study group, exposed to the intervention; SCC: squamous cell carcinoma; tds: three times a day; WHO: World Health Organization severity grading scale for oral mucositis; VAS: Visual Analogue Scale for pain assessment; VHNSSv2: Vanderbilt Head and Neck Symptom Survey, version 2.
